# Future Directions in the Research on Unemployment: Protean Career Orientation and Perceived Employability Against Social Disadvantage

**DOI:** 10.3389/fpsyg.2021.701861

**Published:** 2022-01-24

**Authors:** Chiara Panari, Michela Tonelli

**Affiliations:** ^1^Department of Economics and Management, University of Parma, Parma, Italy; ^2^Department of Humanities, Social Sciences and Cultural Industries, University of Parma, Parma, Italy

**Keywords:** unemployment, protean career orientation, employability, career planning, job search strategies

## Introduction

The level of uncertainty and fear introduced by COVID-19 pandemic has threatened the relationships, work and meanings of existence.

From the point of view of the labor market, the COVID-19 crisis has undermined the illusion of security at work, leading to a massive career shock and accentuating the existing inequities in the labor market, with severe economic and societal implications in terms of career experiences, job opportunities and career paths (Akkermans et al., 2020). During a pandemic, the loss of employment opportunities represents a source of fear which aggravates the intense concerns and anxieties about health and death.

According to a preliminary report from the International Labor Organization (ILO., 2020) estimating between 5.3 and 24.7 million unemployed, the most negative impact will be felt by low-wage and low-skill employees. Jobless individuals tend to be those who have had precarious jobs in fields that typically do not offer long-term contracts, decent wages, and health benefits (ILO., 2020).

Since the individuals' work-lives represents a source of motivation, expression of personal believes and high-quality interpersonal interaction (Crayne, 2020), reconstructing life after this pandemic will need to consider a new perspective of work as a core value in creating decent and decorous work, which has been limited by COVID-19 crisis (Blustein and Guarino, 2020).

This situation has leading researchers to ask questions about the processes by which individuals cope with a job loss experience and the mechanisms triggering attitudes of resilience and exploration of sustainable careers that would imply seeing oneself either in a constantly evolving path, or developing additional skills, or retooling for other jobs and building new career networks (Hite and McDonald, 2020). Studying these aspects will help direct active labor policy interventions aimed at promoting and supporting the employability of people looking for work.

## The Literature on Unemployment

Most literature has focused on the negative effects of job loss on well-being, such as physiological symptoms, depression and suicide (McKee-Ryan et al., 2005; Paul and Moser, 2009; Wanberg, 2012), limited to the examination of the influence of stress, response, and coping with the results of one's job loss (Gowan, 2014). This is also reflected in the social negative evaluation of being unemployed and the stigmatization of personal weaknesses of the unemployed, which in turn lead to less sympathy, and finally to disadvantaged hiring decisions (Monteith et al., 2016).

In fact, from the point of view of the dominant outgroup represented by employed persons, the stigmatization of unemployment status influences recruiters, hiring managers, and interview panelists in the decision to not hire an unemployed worker. Unemployment status as a social identity is shamed, as with other stigmatized social groups, and psychological processes associated with social identity and stigma contribute to the discrimination (Norlander et al., 2020). Particularly, people who possess system-justifying beliefs are more likely to judge unemployed and their deservingness negatively. Beliefs in a just world are likely to affect negative judgments of an unemployed person's competences (Monteith et al., 2016).

From the point of view of the unemployed themselves, the social stigma of the unemployed as being unmotivated, depressed and without professional abilities or personal resources can generate feelings of weakness and blushing on jobless people, and may in turn negatively impact social connections (Grimmer, 2016). McFadyen (1995) argued that the coping processes used by unemployed people to face this stigma could be influenced by whether they categorized themselves as unemployed or adopt some other categorization.

The social identity approach sustained that social image that arises from group memberships has important consequences for how people view and feel about themselves, and also how they are viewed and evaluated by others. If social identities do not provide positive resources for group members, this negatively reflects on individual self-esteem and well-being (Jetten et al., 2017).

The researches that have focused on the attributional processes used by jobless individuals to explain their condition are heterogeneous and also COVID-19 crisis seems to have altered these processes.

On the one side, unemployment is an undesirable and uncontrolled event and there is an ample literature focused on this view. In this sense, from the unemployed person's point of view on his/her perception of social disadvantage, some studies showed that jobless individuals generally show a greater empathy with unemployed people and attribute unemployment to environmental, rather than personal, factors (Furåker and Blomsterberg, 2003; Van Oorschot and Meuleman, 2014). They seem to justify their situation as a painful experience beyond their control. Consistent with a social identity theory perspective, some authors underlined that jobless individuals use both intragroup and intergroup comparisons and these processes were related to their self-esteem. In period of very high unemployment, like the current one where the stigmatization is less pronounced because external causes are attributed to unemployment, the perception of being similar to the unemployed group (at the intra-group level) enhanced feelings of self-worth. However, greater perceived differences between unemployed people and employers were associated with reduced self-esteem (Sheeran et al., 1995). This finding supports the view that feelings of self-worth are contingent, at least in part, on the perceived status of one's own group relative to other groups (Sheeran et al., 1995).

On the other side, there is also evidence that unemployed people do not share similar experience of unemployment (Creed et al., 2001). (Creed and Evans, 2002) highlight the importance of individual differences when considering the psychological impact of unemployment. In fact, some researchers have found that jobless people hold a stronger prejudices and stigma on unemployed individuals than do employed individuals, especially regarding overall value, ability, motivation, and mental health (Takahashi et al., 2015).

In addition, a few studies on the process of in-group identification showed that the unemployed identified little with their own disadvantaged category, which was perceived as a group to distance themselves from (Wahl et al., 2013). In this sense, unemployed could carry out a process defined by literature as self-group distancing that represents an individual mobility response to dissociate from their stigmatized in-group and avoid the negative experience of being stigmatized (Van Veelen et al., 2020).

Other studies have underlined that the process of in-group identification seemed to be more related to the personal stress one experienced (Ybema et al., 1996), or to family-extended employment (Curtis et al., 2016), or to length of time they are unemployed (Cassidy., 2001), rather than to a comparison between social categories characterized by different statuses. In terms of effects of self-categorization on social support, locus of control and problem-solving, previous experience of unemployment plays a crucial role (Cassidy., 2001). In a Danish study (Pultz and Mørch, 2015), researchers showed that some jobless individuals challenge the traditional representation of the unemployed and describe them as innovative, skilled and able to cope with economic insecurity even though it is stressful. These authors take up the concept of strategic self-management, which refers to a pro-active career orientation.

The identity of “unemployed” can be perceived as flexible and transient, and how person adopts this identity has implications for the person's core cognitive beliefs that influence person's ability to adapt to career events (Thompson et al., 2017). The possibility of perceiving one's unemployment status only as a phase of one's working career and not as a condition of a stigmatized social group could be due to the perception of the permeability of the boundaries between groups of unemployed and employed people. Probably even today, in a situation of large-scale emergency crisis, the boundaries between employed and unemployed people are still much less clear and the perception of failings, poor competencies and welfare stigma previously attributed to the unemployed has changed consistently. In fact, from the out-group point of view, in the HR selection process evaluators tend to have less bias toward unemployed individuals because unemployment has become today a vast and global scale phenomenon (Suomi et al., 2020).

Also from the in-group perspective, unemployment is now much more seen as a temporary phase of the career path rather than a fixed social category. Rather than justifying the system that excluded them from the productive world, which is an attributional process that usually characterizes employed workers in their perception of unemployed category (Monteith et al., 2016), some employees who have lost their job seem to be more engaged in coping with the resulting change and the discontinuity of their working life.

The framework of a career planning concept and career paths over time (Wanberg, 2012) could be considered as yet another approach through which it is possible to examine job loss, by pointing out the dynamic career planning activities over the course of one's unemployment. Furthermore, research focusing on career exploration during the unemployment conditions following a job loss, has the potential to reconsider and change the meaning of job loss to individuals (Zikic and Klehe, 2006).

Our contribution moves in this direction, as it explores some constructs that can influence the perception of unemployment directly from people experiencing job loss, and could be the precursor to a more realistic interpretation of the condition of social disadvantage, thus promoting a more proactive attitude toward job reintegration.

Particularly, we will focus on protean career orientations that play a pivotal role in the search for growth opportunities within the job loss transition and that help people to face, not only the negative factors associated with their situation of uncertainty in connection with the crisis of their professional project, but also to re-evaluate their wider life goals and career paths (Waters et al., 2014).

The protean career concept is strictly related to the employability that refers to “individual's beliefs about the possibilities of finding new, equal, or better employment” (De Cuyper et al., 2011). It arises from a combination of knowledge, practical skills and abilities that individuals develop over the course of their working life in order to achieve their career path, allowing them to make sense of their previous professional experiences and to explore new opportunities (Fugate and Kinicki, 2008).

## The Protean Career Orientation and Perceived Employability as Key Strategies for Work Reintegration

Current literature on unemployment emphasizes how the success of one's job search depends on the sense of individual responsibility and the desire for self-fulfillment in guiding one's career choices, as well as individual beliefs about the possibility of achieving one's goals. In this sense, the concept of Protean Career Orientation (PCO) refers to one's attitude toward career choices, based on the search for self-realization. This attitude implies that an individual is responsible for managing his/her own career and for making career-related decisions shaped on personal values, rather than labor market demands (Briscoe and Hall, 2006).

The two aspects of a protean career orientation are: being self-directed and being value-driven. Self-direction refers to the degree to which an individual has control over his/her own career (Mirvis and Hall, 1994). The aspect of value-driven places career decisions as closely linked to one's own personal values, rather than one being driven by categories of the social system (Briscoe and Hall, 2006). As underlined by Lysova et al. (2015), the sense of meaning that workers derive from work, however, is impacted by work values, understood as the end states people desire and feel they ought to be able to realize through working (Nord et al., 1990). People who show a high level of intrinsic values, as freedom and self-growth, has an higher protean career orientation and defines career success in terms of psychological factors as compared with traditional career; protean career orientation is also focused on continuous learning in professional development (Hall, 2004).

One of the critical aspects connected with the state of unemployment is the perception of uncontrollability, which can lead one to focus on external factors and to feel closer to other social disadvantaged groups (Bukowski et al., 2019), rather than to focus on internal motivational resources. On the other hand, in the context of unemployment, the protean career orientation activates a reverse process of reworking one's career path, offering a different interpretation of one's social condition, because the person focuses on his/her aspirations and goes back to feeling like he/she still has the personal resources to invest in a new professional project. The prerequisite for a protean career attitude is the overcoming of the categorization and evaluation imposed by the external social world, because those values are founded on the notion of career actors—as opposed to organizations—who take responsibility of their own careers (Hall, 2002). Protean people seem to have more internal control over their career path and this is in line with unemployment research, that underlined the role of internal LOC in predicting reemployment (Meyers and Houssemand, 2010). Applying the perspective of the social determination theory to unemployment, some authors (Vansteenkiste et al., 2005) found that perception of being forced to search for a job, moving by controlled motivation accompanied by stressful and pressuring experiences, negatively predicted their general health. On the contrary, if unemployed perceive the search for a job as an autonomous and personal choice because employment is seen as an opportunity to develop their skills, they have an internal motivation that enhance behavioral effectiveness, greater volitional persistence, and enhanced subjective well-being. This motivational process is the basis of the perception of controllability of the protean orientation. Also social cognitive career theory highlighted the importance of self-regulatory efficacy, which involves beliefs about controlling motivational aspects of the job search, and personal goals, as behavioral intentions to act in ways that produce desired outcomes, in predicting reemployment success (Thompson et al., 2017).

In this sense, when considering re-employment, Waters et al. (2014) emphasized that a protean career orientation helped individuals to clarify and express their goals during unemployment and to find a sense of positive identity (Zafar et al., 2017).

Secondly, another core aspect is related to the loss of self-esteem (Kanfer et al., 2001), that represents a psychological consequence of unemployment. During unemployment PCO may help unemployed people to maintain a positive self-esteem. Protean orientation could be interpreted as a mechanism through which unemployed feel much more similar to people who belong to the world of work and activate a self-group distancing process also for the type of careers that characterize working life. In fact, there were disruptive and macroeconomic factors in the labor market that have changed how individuals conceptualize their careers more fragmented and discontinuous compared to the past (Briscoe et al., 2012).

People who manage their careers from a protean orientation do not link their career identity to the organization and loss. This perception does not lead to the lack of the sense of identity that sometimes occurs after the job loss (Waters et al., 2014). Instead, people with low PCO levels will be less proactive in finding resources for the enhancement of their skills, and their level of self-esteem will likely be lower during the period of unemployment. This can discourage people from looking for a new job, as it affects the belief that they can find it (Hirschi et al., 2017).

Thirdly, people with a high protean career level become more independent and flexible in managing their career opportunities in response to social changes in work organization (Wiernik and Kostal, 2018). In the literature, the concept of protean career has been associated with the concept of boudaryless career which refers to a career characterized by different levels of physical and psychological movement among organizations (Sullivan and Arthur, 2006), which metaphorically recalls the permeability of the boundaries between workers and unemployed. Consequently, high-PCO individuals are in charge of their own career development (Hall et al., 2018) and can adjust to the current dynamic labor market. People with a high PCO tend to: be more learning-oriented; have high self-esteem and clearer goals; and formulate specific career plans (Li et al., 2019).

This proactive attitude translates to a more effective job search during unemployment (Waters et al., 2014). In fact, adopting a protean self-directed approach may lead individuals to regularly explore the situation of work environment in order to increase their chances of finding a job that will help them achieve their personal projects.

Self-managing one's career leads people to become more aware of their acquired professional skills but also increases the knowledge and competencies required in the labor market (Bozionelos and Bozionelos, 2015).

In this sense, recent studies have shown that people oriented toward a protean career are likely to have a high level of perceived employability (Baruch et al., 2019; Cortellazzo et al., 2020).

The perceived employability is the second key construct that plays a central role in managing one's work history in unemployment conditions.

When considering changes in career development and paths, increasing one's employability is an important task for both the unemployed and those seeking new employment, as their career may depend on perceived employability.

Employability has been studied mainly from three perspectives. Fugate and Kinicki (2008) proposed a dispositional approach to employability which identifies a range of traits (for example, openness to change, proactivity, and resilience), that facilitates proactivity in adapting to work and career environments. Van Der Heijde et al. (2006) elaborated a competence-based conceptualization of employability, in which the dimension of occupational expertise is complemented with four general competences: anticipation and optimization, personal flexibility, corporate sense and balance. The authors distinguish between two different types of adaptation to changes in the internal and external labor market, the first one that is referred to as anticipation and optimization, and one more passive variant entitled personal flexibility. The concept of corporate sense refers to participation and performance in different workgroups, such as the department, working teams, occupational community or other networks. Finally, balance is defined as compromising between opposing employers' interests as well as one's own opposing work, career and private interests. Finally, the third perspective focuses on perceptions of employability which Vanhercke et al. (2014) define as the individual's perceptions of possibilities of obtaining and maintaining employment.

In the field of unemployment, we refer to the third perspective concerning external perceived employability, that has been also defined by Berntson et al. (2006) as the subjective individual perception of the ability to evaluate one's skill at getting a job. In this sense, employability represents the perception of employment opportunities with the current employer or with another employer (Rothwell and Arnold, 2007; De Cuyper and De Witte, 2008). The subjective perception, in fact, of being able to relocate to the professional world had a strong motivational impact, which in turn affected the implementation of realistic assessments of one's actual possibility of relocation and the use of functional strategies to achieve one's professional goals (Van den Broeck et al., 2010), such as skill development (De Vos et al., 2011; Vanhercke et al., 2014).

Furthermore, perceived employability increases the feelings of control over careers and job search activities, and it is related to a minor duration of unemployment, and to re-employment (Consiglio et al., 2021).

Research also showed that perceived employability could help mitigate the negative effects of job loss, such as emotional implications (Hodzic et al., 2015; Consiglio et al., 2021).

In the context of job loss, individuals who are more employable will perceive less impairment from the job loss, will engage in more job search activity and will achieve higher quality reemployment (Fugate et al., 2004). Koen et al. (2013) showed that employability also increased long-term reemployment opportunities (McKee-Ryan et al., 2005; Paul and Moser, 2009; Lim et al., 2016; Lo Presti and Pluviano, 2016). Perceived employability could represent an individual's belief that reduces the differences with the people who are in the job market because it focuses on the perception of one's personal skills and opportunities for change affecting proactive behaviors and cognitive reinterpretation of job loss. According to social identity theory, especially if boundaries between groups are perceived more permeable, protean career orientation and perceived employability could be seen as an individual mobility strategy to distance from a devalued social group and achieve more positive social identities.

Protean individuals who see themselves as more employable are less likely to feel as they are part of a stigmatized category allowing to protect themselves from social stigma, even if the stigma consciousness of employment does not always have negative consequences in terms of proactivity (Krug et al., 2019) especially in in the context of the COVID-19 health crisis. A high levels of protean career orientation and perceived employability allow to evaluate the experience of unemployment differently and this approach leads jobless individuals to believe in the future. In fact, their perception of available opportunities in the labor market may be selective and more engaged in targeted research (Zakkariya and Nimmi, 2021).

## Conclusion

As a career shock, the COVID-19 crisis has led us to develop new studies to identify and implement targeted actions that could contribute not only to improving the general well-being of unemployed persons, but also to increasing their likelihood of finding work.

In the actual socio-economic context characterized by a general lack of job opportunities, and considering the diffusion of new career paths characterized by frequent work changes and transitions, our question is: “Are the unemployed still stigmatized or do they perceive themselves to be a disadvantaged category today?”.

Following the economic consequences of the pandemic, the social perception of unemployment has changed, limiting prejudices against jobless people by employed individuals. This could have an impact on the unemployed perception of their work condition. Unemployed people should therefore suffer a lesser loss of the sense of self-esteem and self-efficacy and rely on their own proactivity to find a new job. To be successful in finding employment a person must believe they have the skills and abilities to do so. In this sense, gaining deeper understanding of the role of a protean career orientation and of perceived employability can offer unemployed people new ways to create change for themselves. In fact, people with a high level of protean career and employability are less likely to feel that they are part of a disadvantaged category, have a high self-esteem and self-efficacy, as they evaluate their experience of unemployment differently and this approach activates proactive behavior in preparatory and active job search.

Even in the case of unemployed individuals seeking guidance and advice to support their return to the labor market, protean people with a high level of perceived employability tended to better estimate their skills and better define their professional goals by identifying possible perspectives for getting out of the unemployed group in which they do not recognize themselves.

In terms of career counseling, working with unemployed clients should focus on building positive perspectives in connection with the clients' career goals and their sense of self direction and responsibility in order to promote control over their career paths. In fact, people with high levels of PCO are less identified in a disadvantaged social category, and this aspect could be used during the counseling to modify the cognitive interpretation of the unemployment status and promote proactivity and agency. In this sense, a counseling centered on protean career orientation and perceived employability should be compared to the develop of proactive coping strategies. Counselors should help people to evaluate the period of unemployment as an opportunity to redefine professional goals in a flexible way and develop a plan for achieving them. For example, starting by the reflection on the pandemic situation in terms of changed traditional working methods and roles, counseling can be viewed as a chance to invest in training and updating one's skills, to respond to a significantly changed labor market, especially from the point of view of digital skills. High PCO and perceived employability represent a great motivational and emotional investment in job search that can help to reach job goals, but it may happen that unemployed have to face difficulties and failures in job search. In this sense, a high PCO allows people to collect informations and reflect about their skills, and make plans based on realistic and objective opportunities. Through this step of research and evaluation, people should gain self-awareness and define achievable goals and evaluate alternatives in case of failure, protecting themselves, partially, from emotional negative consequences.

Furthermore, when the protean career orientation is adopted, employability is more effectively used in job searching, because unemployed become more aware of their values, projects, technical and soft skills and develop proactive career strategies (Panari et al., 2020). This perspective can maintain a positive sense of personal professional identity whilst focusing on solutions to get out of the social disadvantage, rather than on the causes of the unemployment situation.

## Author Contributions

All authors listed have made a substantial, direct, and intellectual contribution to the work and approved it for publication.

## Conflict of Interest

The authors declare that the research was conducted in the absence of any commercial or financial relationships that could be construed as a potential conflict of interest.

## Publisher's Note

All claims expressed in this article are solely those of the authors and do not necessarily represent those of their affiliated organizations, or those of the publisher, the editors and the reviewers. Any product that may be evaluated in this article, or claim that may be made by its manufacturer, is not guaranteed or endorsed by the publisher.
